# Sequential Treatments with Tongsai and Bufei Yishen Granules Reduce Inflammation and Improve Pulmonary Function in Acute Exacerbation-Risk Window of Chronic Obstructive Pulmonary Disease in Rats

**DOI:** 10.1155/2016/1359105

**Published:** 2016-08-01

**Authors:** Xiaofan Lu, Ya Li, Jiansheng Li, Haifeng Wang, Zhaohuan Wu, Hangjie Li, Yang Wang

**Affiliations:** ^1^Dongzhimen Hospital, Beijing University of Chinese Medicine, Beijing 100700, China; ^2^Institute for Geriatrics, Henan University of Chinese Medicine, Zhengzhou, Henan 450046, China; ^3^Collaborative Innovation Center for Respiratory Diseases Diagnostics, Treatment and New Drug Research and Development in Henan Province, Zhengzhou, Henan 450046, China; ^4^Institute for Respiratory Diseases and the Level Three Laboratory of Respiration Pharmacology of Chinese Medicine, the First Affiliated Hospital, Henan University of Chinese Medicine, Zhengzhou, Henan 450000, China; ^5^Central Laboratory, the First Affiliated Hospital, Henan University of Chinese Medicine, Zhengzhou, Henan 450000, China; ^6^Department of Respiratory Diseases, the First Affiliated Hospital of Henan University of Chinese Medicine, Zhengzhou, Henan 450000, China

## Abstract

*Background*. Sequential treatments of Chinese medicines for acute exacerbation of chronic obstructive pulmonary disease (AECOPD) risk window (RW) have benefits for preventing reoccurrences of AEs; however, the effects on pulmonary function, pulmonary, and systemic inflammatory biomarkers remain unclear.* Methods*. Cigarette-smoke/bacterial infections induced rats were randomized into Control, COPD, AECOPD, Tongsai Granule/normal saline (TSG/NS), moxifloxacin + salbutamol/NS (MXF+STL/NS), TSG/Bufei Yishen Granule (BYG), MXF+STL/STL, and TSG+MXF+STL/BYG+STL groups and given corresponding medicine(s) in AE- and/or RW phase. Body temperature, pulmonary function, blood cytology, serum amyloid A (SAA) and C-reactive protein (CRP), pulmonary histomorphology and myeloperoxidase (MPO), polymorphonuclear (PMN) elastase, interleukins IL-1*β*, IL-6, and IL-10, and tumor necrosis factor- (TNF-) *α* expressions were determined.* Results*. Body temperature, inflammatory cells and cytokines, SAA, CRP, and pulmonary impairment were higher in AECOPD rats than stable COPD, while pulmonary function declined and recovered to COPD level in 14–18 days. All biomarkers were improved in treated groups with shorter recovery times of 4–10 days, especially in TSG+MXF+STL/BYG+STL group.* Conclusion*. Sequential treatments with Tongsai and Bufei Yishen Granules, during AECOPD-RW periods, can reduce inflammatory response and improve pulmonary function and shorten the recovery courses of AEs, especially the integrated Chinese and Western medicines.

## 1. Background

Chronic obstructive pulmonary disease (COPD) is commonly accompanied by acute exacerbations (AEs), which significantly contribute to morbidity and mortality [1]. Acute exacerbations are usually caused by pathogen infection-related inflammation and other insults. Proinflammatory stimuli in the lung recruit inflammatory cells, such as neutrophils, eosinophils, macrophages, and lymphocytes. These cells can secrete proinflammatory cytokines, chemokines, and proteases, leading to the destruction of pulmonary parenchyma and remodeling of multiple components of airway epithelium and contributing to the pathogenesis of AECOPD and the development of emphysema [2, 3]. Previous studies have shown that the concentrations of C-reactive protein (CRP), interleukins IL-6 and IL-1, tumor necrosis factor- (TNF-) *α*, and myeloperoxidase (MPO) and the number of polymorphonuclear (PMN) cells are positively correlated with the severity and poor prognosis of AECOPD [4–7]. However, in the subsequent remission stage, inflammatory indicators presented unstable trends. The numbers of white blood cells (WBCs) and neutrophils in the sputum and blood were increased 24 h after infection and were significantly decreased 3 days after the patients received medication, but the numbers did not fully recover until 10–40 days after infections [8–12]. This unstable period is defined as the AECOPD risk window (RW), which begins approximately 7–21 days after exacerbation in AECOPD patients but does not recover to the baseline of the stable phase and is characterized by decreased body temperature, incompletely recovered pulmonary function, decreased inflammation, and increased risk of subsequent exacerbations. The recurrences of AEs during this period may require readmissions and may increase mortality [13]. A similar variable period was also observed in a sequential COPD-AE-RW rat model, where the levels of inflammatory indicators, such as the number of WBCs and neutrophils and serum amyloid A (SAA) and CRP levels, varied rapidly for 5 days in the AE phase, but the changes were mild in the subsequent 10 days of the RW phase [14, 15].

In traditional Chinese medicine (TCM), COPD is classified as FEIZHANG disease, for which the treatments are based on syndrome differentiation. At different stages of AE and stable phases of COPD, the syndromes are completely different [16]. Generally, the primary syndrome in the AE period is phlegm-heat obstructing lungs, whereas lung-kidney qi deficiency is present in the stable phase. In the risk window period, pathogenesis is presented as a syndrome of lingering pathogen infection due to a deficiency in vital qi, in which the qi deficiency is superior to the excess pathogenic syndrome and is characterized by alleviated clinical symptoms, incomplete recovery of pulmonary function, and high risk of AE recurrence and rehospitalization [17]. Clinically, the method of clearing heat and dissipating phlegm is applied to the phlegm-heat obstructing lung syndrome, which is mainly induced by bacteria and/or viruses and is characterized by fever, cough, and spitting yellow phlegm, as well as pharyngalgia and chest distress [18, 19]. For the stable phase and risk window period, the main treatment principle is to reinforce the deficiency in vital qi because most or all of the excess syndromes have disappeared. In previous studies, Tongsai Granule was confirmed to clear heat-phlegm, relieve cough and breathlessness, and depress the systemic inflammation in AECOPD patients, including serum IL-1*β*, IL-6, and IL-8 levels [20, 21]. Furthermore, it also inhibited the expression of matrix metalloproteinases MMP-2 and MMP-9, type III precollagen (PCIII), transforming growth factor- (TGF-) *β*, laminin (LN), and hyaluronic acid (HA) in AECOPD rats [22, 23]. Bufei Yishen Granule was also shown to improve lung function and reduce the incidence and duration of AE in COPD patients after a 6-month treatment and even in a 12-month follow-up without treatment [24]; the results were confirmed in a rat model [25, 26].

In this study, we attempted to explore the effects of sequential treatments with Tongsai Granule (TSG) and Bufei Yishen Granule (BYG) in the AE-RW period in a rat model by observing improvements in pulmonary function, inflammatory biomarker levels, and pulmonary histomorphology.

## 2. Methods

### 2.1. Animals

Thirty-two male and 32 female 2-month-old Sprague-Dawley rats, weighing 200 ± 20 grams (g), were provided by the Experimental Animal Center of Henan Province (Special Pathogen Free, SCXK (Henan) 2005-0001) and accommodated in individual ventilated cases for 7 days in the facility in the First Affiliated Hospital, Henan University of Traditional Medicine, Zhengzhou, Henan, China, before experiments were performed. The room temperature was maintained at 25 ± 1°C, the relative humidity was 50 ± 10%, with 10 to 15 gas changes per hour, the ammonia concentration was ≤14 mg/m^3^, and the noise was ≤60 db. The rats had free access to sterilized feed and water.

### 2.2. Cigarette

Hongqi Canal® Filter cigarettes (tobacco type, tar 10 mg, nicotine content 1.0 mg, and carbon monoxide 11 mg) were provided by Henan Zhongyan Industry Company (Zhengzhou, Henan).

### 2.3. Bacteria

*Klebsiella pneumoniae* (KP; strain: 46114) was provided by the National Center For Medical Culture Collections (Beijing, China) and was prepared at a concentration of 6 × 10^8^ and 6 × 10^14^ colony forming units (CFU) per milliliter (mL) in suspension before bacteria challenges.

### 2.4. Drugs

Tongsai Granule consists of Ting Li Zi (*Lepidium apetalum* Willd.) 12 g, Di Long (*Pheretima aspergillum* (E. Perrier)) 12 g, Chuan Bei Mu (*Fritillaria cirrhosa* D. Don) 12 g, Da Huang (*Rheum officinale* Baill.) 6 g, Ma Huang (*Ephedra sinica* Stapf.) 9 g, Chi Shao (*Paeonia anomala* subsp.* veitchii* (Lynch) D. Y. Hong and K. Y. Pan) 12 g, Mai Dong (*Ophiopogon japonicus* (Thunb.) Ker Gawl.) 12 g, and Ai Di Cha (*Ardisia japonica* (Thunb.) Blume) 15 g [22]. Bufei Yishen Granule consists of Ren Shen (*Panax ginseng* C. A. Mey.) 9 g, Huang Qi (*Astragalus membranaceus* (Fisch.) Bunge) 15 g, Gou Qi (*Lycium chinense* Mill.) 12 g, Shan Zhu Yu (*Cornus officinalis* Siebold and Zucc.) 12 g, Yin Yang Huo (*Epimedium rotundatum* K. S. Hao) 9 g, Wu Wei Zi (*Schisandra chinensis* (Turcz.) Baill.) 9 g, and Ai Di Cha (*Ardisia japonica* (Thunb.) Blume) 9 g [27]. These drugs were prepared by the Department of Pharmacology in the First Affiliated Hospital, Henan University of Chinese Medicine, Zhengzhou, China. Moxifloxacin (MXF) hydrochloride tablets (0.4 g/tablet, Bayer, Germany) and salbutamol (STL) sulfate tablets (2 mg/tablet, Yabang, Jiangsu, China) were crushed and prepared as 10 mg/mL and 1 mg/mL solutions, respectively, before administrations.

### 2.5. Model Preparation

**Figure 1:**
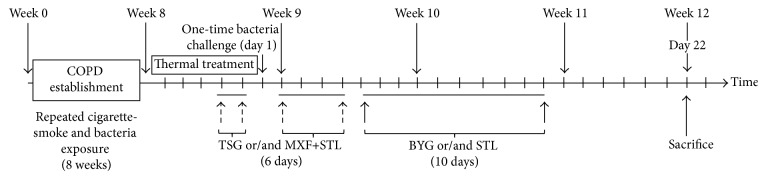
Experimental flow and key time points for the administrations. Week 1 through week 8: COPD model preparation period. Day 1: the rats were challenged with* Klebsiella pneumonia* solution (6 × 10^14^ CFU/mL) after a 5-day thermal treatment. Tongsai Granule (TSG) or/and moxifloxacin (MXF) were administered to the AECOPD rats 2 days before and 4 days after challenge. Bufei Yishen Granule (BYG) and/or salbutamol (SLT) were administered over the next 10 days. The rats were sacrificed at the end of week 12 (Day 22). BYG: Bufei Yishen Granule; COPD: chronic obstructive pulmonary disease; MXF: moxifloxacin; RW: risk window; TSG: Tongsai Granule; STL: salbutamol.

After adaptive accommodation for 7 days, the COPD model was established by cigarette-smoke and KP exposure, as previously reported [28]. The rats were housed in a sealed chamber and exposed to tobacco smoke (3,000 ± 500 parts per million (ppm)) generated by a smoke machine (BUXCO, NC, USA) for two 30-minute exposures per day for 8 weeks, with three-hour intervals. A KP solution prepared at 6 × 10^8^ CFU/mL was slowly dropped into both nostrils in an alternating fashion at 0.1 mL/animal every 5 days for 8 weeks. The AECOPD rat model of phlegm-heat syndrome was established at week 9 according to previous reports [15, 29]. In the first 5 days of week 9, the rats were exposed to a heated ventilated chamber (39.0 ± 0.5°C) twice for 30 min at three-hour intervals. They were then intratracheally challenged with the KP solution (0.1 mL/animal, 6 × 10^14^ CFU) on the 6th day of week 9 (Day 1) after being anesthetized with chloral hydrate (0.28 g/kg body weight). All animals were sacrificed on Day 22 (Figure 1).

### 2.6. Grouping and Administrations

Sixty-four rats were randomized into Control, COPD, AECOPD, TSG/normal saline (TSG/NS), MXF+STL/NS, TSG/BYG, MXF+STL/STL, and TSG+MXF+STL/BYG+STL groups using a random number table (4 males and 4 females per group). Rats were administered intragastrically according to the protocol presented in Table 1 from the 4th day (Day −1) of week 9 to Day 16, excluding Day 1 (the challenge day). The sequential treatments with Western medicine were designed according to the “Global Strategy for the Diagnosis, Management, and Prevention of Chronic Obstructive Pulmonary Disease”* (update 2014)* [16].

The equivalent doses of TSG (7.2 g/kg/d), BYG (4.44 g/kg/d), MXF (27 mg/kg/d), and STL (0.41 mg/kg/d) were calculated using the following formula according to published references: *D*_rat_ = *D*_human_ × (*K*_rat_/*K*_human_)×(*W*_rat_/*W*_human_)^2/3^; *D*: dose; *K*: body shape index; *K* = *A*/*W* ^2/3^ (*A*: surface area/m^2^, *W*: body weight/kg); *W*: body weight [30].

### 2.7. General Status

Body weights were recorded on weeks 4 and 8 and then weekly from week 9 to week 12.

### 2.8. Pulmonary Function Tests

Peak expiratory flow (PEF) was measured with an unrestrained Whole Body Plethysmograph (uWBP) system (Buxco, NY, USA) at the end of week 4 and week 8 and each weekend from week 9 to week 12. Forced expiratory volume 0.3 s (FEV0.3) and forced vital capacity (FVC) was determined with a FinePoint*™* Pulmonary Function Test system (Buxco, NY, USA) on Day 22 after the animals were anesthetized and prior to sacrifice.

### 2.9. Blood Cytological Analysis and Serum Inflammatory Biomarkers Detection

The numbers of white blood cells (WBCs), neutrophils, monocytes, and lymphocytes in tail vein blood were analyzed with a hemocyte analyzer every 2 days from Day 0 to Day 22. CRP and SAA levels were also detected in the serum of the vein blood by enzyme-linked immunosorbent assay (ELISA) (Boster, Wuhan, China).

Whole blood was collected from the aorta abdominalis after the animals were anesthetized and sacrificed on Day 22. MPO, PMN elastase, IL-1*β*, IL-6, IL-10, and TNF-*α* levels in the serum were detected by ELISA (Boster, Wuhan, China).

### 2.10. Lung Tissue Sectioning and Bronchoalveolar Lavaging

All animals were sacrificed by exsanguination of the abdominal aorta after blood was collected. The trachea was cannulated, and the heart/lung block was removed from the thoracic cavity. The right extrapulmonary bronchus was ligated with sutures, and the right lung lobes were removed. The left lung lobe was lavaged with normal saline, and the recovered bronchoalveolar lavage fluid (BALF) was used to determine the total cell number, numbers of specific cell types, and cytokine levels. The lavaged left lung lobe was perfusion-fixed with 10% neutral buffered formalin via the trachea at a constant pressure of 30 cm fixative for 2 h, and it was immersed in the same fixative for at least 24 h before further processing.

### 2.11. BALF Cytological Analysis and Inflammatory Biomarkers Detection

Total cell numbers were determined manually using a hemocytometer, and the numbers of different cell types, such as neutrophils, macrophages, and lymphocytes, were determined under inverted and upright microscopes (Olympus, Japan). The left BALF was centrifuged, and the supernatant was collected to determine IL-1*β*, TNF-*α*, IL-6, IL-10, MPO, and PMN elastase levels by ELISA (Boster, Wuhan, China).

### 2.12. Pulmonary Morphology and Morphometry

Randomly orientated, serial sections of the formalin-fixed left lung lobe were processed using routine methods and embedded in paraffin. The tissue slices (4 *μ*m) were deparaffinized and stained with hematoxylin-eosin (HE) for histopathology. The slides were blinded, and the alveolar cavity and density of alveoli were determined as follows: Mean linear intercept (MLI) (*μ*m) = *L*/*N*_s_. After a cross (+) was drawn through the center of each photo, the number of alveolar septa (*N*_s_) lying on the cross was counted, and then the total length of the cross (*L*) was measured: mean alveolar numbers (MAN) (/mm^2^) = *N*_a_/*A*. The number of pulmonary alveoli in each visual field (*N*_a_) and the area of the visual field (*A*) were measured [31].

### 2.13. Statistical Analysis

The data are presented as the means ± standard errors (SE). Chi-square test was applied to the mortality data. For repeated measurements, such as body weight, body temperature, cytological analysis, SAA and CRP levels, and PEF, repeated measures of a general linear regression equation were applied. One-Way ANOVA was applied to the FEV0.3, FVC, FEV0.3/FVC, levels of inflammatory factors in the BALF and serum, and pulmonary morphometry results. Statistical analyses were performed with SPSS Statistics 19.0 software (IBM, CA, USA). A two-tailed *P* < 0.05 indicated statistical significance.

## 3. Results

### 3.1. Mortality

Two rats in COPD and AECOPD groups died as a result of pulmonary abscesses during the preparation period of the COPD model. Another rat in AECOPD group died for the same reason on Day 3, 48 h after bacterial challenge (Table 2).

### 3.2. Body Weight

**Figure 2:**
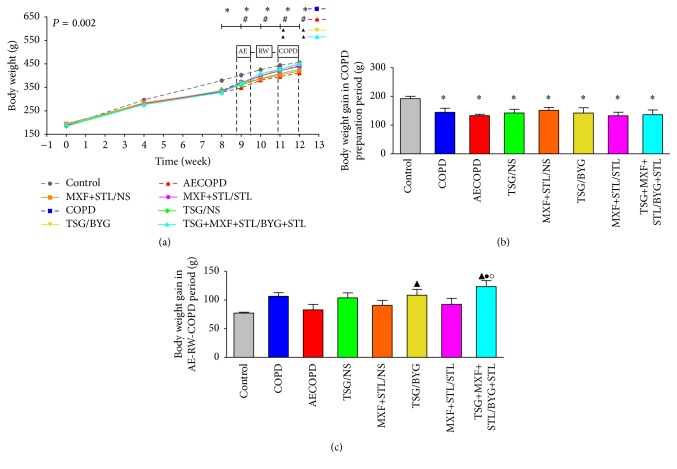
Temporal changes in body weights (a) over the 12-week experimental period; body weight gain in COPD preparation period (b) and AE-RW-COPD periods (c) in each group. AE: acute exacerbation; BYG: Bufei Yishen Granule; COPD: chronic obstructive pulmonary disease; MXF: moxifloxacin; NS: normal saline; RW: risk window; TSG: Tongsai Granule; STL: salbutamol. *N* = 6. Repetitive measurement deviation analysis of body weights: *P* = 0.002. ^*∗*^ *P* < 0.05, versus Control group; ^#^ *P* < 0.05, versus the COPD group; ^▲^ *P* < 0.05, versus AECOPD group; ^●^ *P* < 0.05, versus TSG/BYG group; ^○^ *P* < 0.05, versus MXF+STL/STL group.

As shown in Figure 2, the body weights of COPD rats were decreased from week 8 to week 12 compared with Control group (*P* < 0.05). After challenge with the KP solution, body weights of AECOPD group decreased from week 9 to week 12 compared with COPD group (*P* < 0.05). Body weights in the treated groups showed increasing trends after bacteria challenge compared with AECOPD group, and the body weights of TSG/BYG and TSG+MXF+STL/BYG+STL groups were significantly higher than those of AECOPD group at week 11 and week 12 (*P* < 0.05) (Figure 2(a)).

Body weight gain in COPD rats was lower than that in Control rats during COPD model preparation period (*P* < 0.05); it was higher in TSG/BYG and TSG+MXF+STL/BYG+STL groups than in AECOPD group in AE-RW-COPD period, and it was even higher in TSG+MXF+STL/BYG+STL group than in TSG/BYG and MXF+STL/STL groups (*P* > 0.05) (Figures 2(b) and 2(c)).

### 3.3. Body Temperature

**Figure 3:**
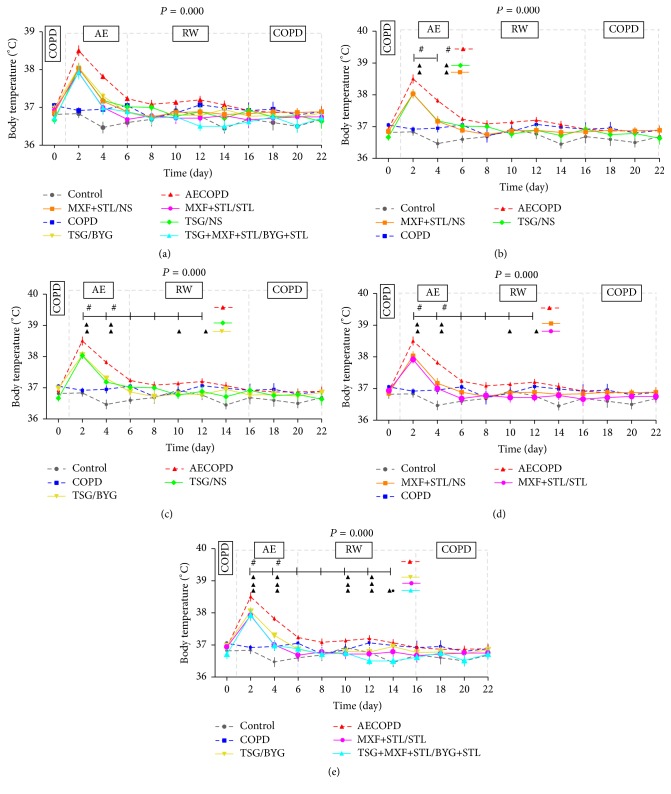
Temporal changes in body temperature (a) in rats administered sequential/nonsequential treatments. Panels (b), (c), (d), and (e) were split from panel (a) and indicate the comparison of sequential and nonsequential treatments with traditional Chinese medicine, Western medicine, or integrated medicines. AE: acute exacerbation; BYG: Bufei Yishen Granule; COPD: chronic obstructive pulmonary disease; MXF: moxifloxacin; NS: normal saline; RW: risk window; TSG: Tongsai Granule; STL: salbutamol. *N* = 6. Repetitive measurement deviation analysis of the body temperatures: *P* = 0.000. ^#^ *P* < 0.05, versus COPD group; ^▲^ *P* < 0.05, versus AECOPD group; ^●^ *P* < 0.05, versus TSG/BYG group. Bacteria challenge was performed on Day 1.

As shown in Figure 3(a), the variations in body temperature in COPD group were approximately the same as those in the Control group. Twenty-four hours after bacterial challenge, body temperatures in AECOPD group increased sharply compared with those in COPD group (*P* < 0.05); the temperatures rapidly decreased over the next 4 days, fluctuated more smoothly in the subsequent days, and finally were synchronized with COPD group on Day 16 (*P* < 0.05). Compared with AECOPD group, body temperatures of the treated groups decreased on Day 2 and sharply decreased over the next 2 days (*P* < 0.05) (Figures 3(b), 3(c), 3(d), and 3(e)). Then, the curve shifted below that of COPD group on Day 6 and presented a mild decline in the subsequent days (Figures 3(b), 3(c), 3(d) and 3(e)). Moreover, the temperatures of TSG+MXF+STL/BYG+STL group were even lower than those in TSG/BYG group on Day 14 (*P* < 0.05) (Figure 3(e)).

### 3.4. Pulmonary Function

**Figure 4:**
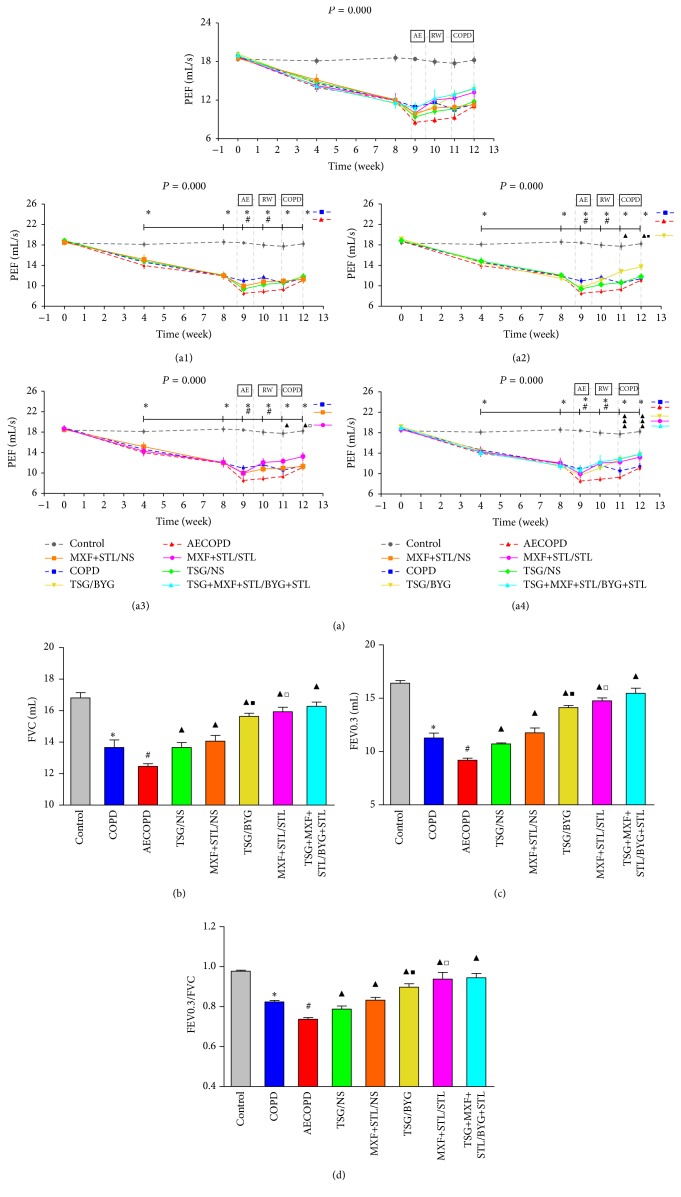
Changes in peak expiratory flow (PEF) (a) and forced vital capacity (FVC) (b), forced expiratory volume at 0.3 s (FEV0.3) (c), and FEV0.3/FVC (d) in rats treated with sequential/nonsequential treatments. Panels (a1)–(a4) from panel (a) indicate the comparisons of the sequential and nonsequential treatments with traditional Chinese medicine, Western medicine, or integrated medicines, respectively. AE: acute exacerbation; BYG: Bufei Yishen Granule; COPD: chronic obstructive pulmonary disease; MXF: moxifloxacin; NS: normal saline; RW: risk window; TSG: Tongsai Granule; STL: salbutamol. *N* = 6. Repetitive measurement deviation analysis of PEF: *P* = 0.000. ^*∗*^ *P* < 0.05, versus Control group; ^#^ *P* < 0.05, versus COPD group; ^▲^ *P* < 0.05, versus AECOPD group; ^■^ *P* < 0.05, versus TSG/NS group; ^□^ *P* < 0.05, versus MXF+STL/NS group. Bacterial challenge was performed on the 6th day of week 9.

PEF in COPD group was significantly lower than in the Control group from week 4 to week 12 (*P* < 0.05) (Figure 4(a)). PEF was significantly reduced in AECOPD group 24 hours after bacterial challenge (*P* < 0.05), showed an increasing trend over the next 3 weeks, and recovered to the baseline values of COPD group at week 12. There was a slight increase in PEF in the treated groups compared with AECOPD group beginning at week 9, which returned to the baseline level of COPD group at week 11, approximately 1 week earlier than AECOPD group. PEF was higher in TSG/BYG and MXF+STL/STL groups than in TSG/NS and MXF+STL/NS groups at week 12 (*P* < 0.05).

As shown in Figures 4(b), 4(c), and 4(d), FVC, FEV0.3, and FEV0.3/FVC were decreased in COPD group compared with Control group, respectively (*P* < 0.05), and were substantially decreased in AECOPD group. All of the above-mentioned parameters were higher in the treated groups than in AECOPD group and were much higher in TSG/BYG and MXF+STL/STL groups than in the TSG/NS and MXF+STL/NS groups (*P* < 0.05).

### 3.5. Cell Types in the Peripheral Blood

**Figure 5:**
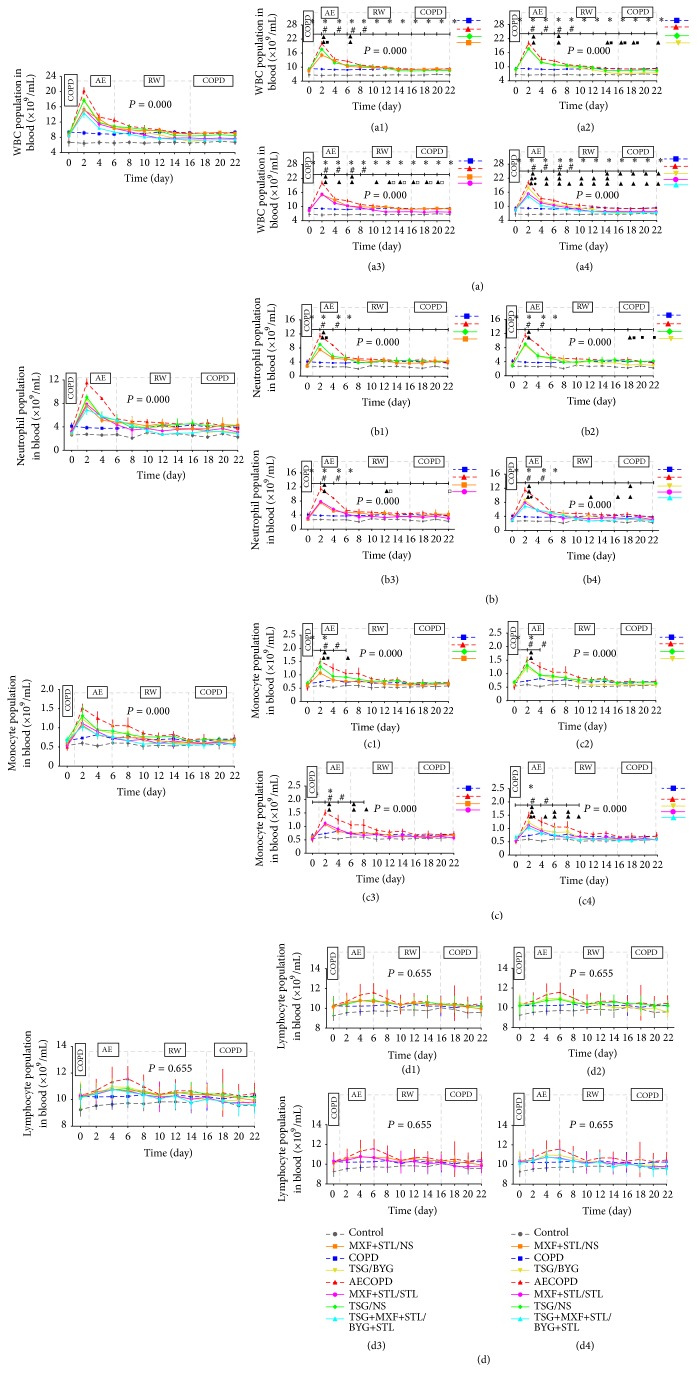
Temporal changes in the numbers of white blood cells (WBCs) (a), neutrophils (b), monocytes (c), and lymphocytes (d) in rats administered sequential/nonsequential treatments. Panels (a1)–(a4), (b1)–(b4), (c1)–(c4), and (d1)–(d4) from panels (a), (b), (c), and (d) indicate the comparisons of sequential and nonsequential treatments with traditional Chinese medicine, Western medicine, or integrated medicines, respectively. AE: acute exacerbation; BYG: Bufei Yishen Granule; COPD: chronic obstructive pulmonary disease; MXF: moxifloxacin; NS: normal saline; RW: risk window; TSG: Tongsai Granule; STL: salbutamol. *N* = 6. Repetitive measurement deviation analysis of the numbers of WBCs, neutrophils, and monocytes: *P* = 0.000; lymphocytes: *P* = 0.655. ^*∗*^ *P* < 0.05, versus Control group; ^#^ *P* < 0.05, versus COPD group; ^▲^ *P* < 0.05, versus AECOPD group; ^■^ *P* < 0.05, versus TSG/NS group; ^□^ *P* < 0.05, versus MXF+STL/NS group; ^●^ *P* < 0.05, versus TSG/BYG group. Bacterial challenge was performed on Day 1.

As shown in Figures 5(a), 5(b) and 5(c), there were more WBCs in COPD group than in Control group throughout the experiment (*P* < 0.05), and the numbers of monocytes and neutrophils were increased in the first 2–8 days (*P* < 0.05). After challenge with the KP solution, the indicators mentioned above were highly elevated in AECOPD group (*P* < 0.05), decreased rapidly over the next 4 days, presented a smooth decreasing trend in the subsequent days, and returned to the baseline levels of COPD group on Day 16. For the treated groups, all indicators were reduced to different extents compared with AECOPD group on Day 2; they declined rapidly over the next 2 days, changed more smoothly in the subsequent days, and returned to the levels of COPD group or decreased further on Days 8–12. In addition, the numbers of WBCs, neutrophils, and monocytes were decreased in MXF+STL/NS group compared with TSG/NS group on Day 2, and the numbers of WBCs in MXF+STL/STL and TSG+MXF+STL/BYG+STL groups were even lower than those in TSG/BYG group (*P* < 0.05). Meanwhile, the numbers of neutrophils and monocytes were significantly reduced in TSG+MXF+STL/BYG+STL group compared with TSG/BYG group (*P* < 0.05). Additionally, the numbers of WBCs in TSG/BYG and MXF+STL/STL groups were reduced compared with TSG/NS and MXF+STL/NS group from Day 12 to Day 20, respectively (*P* < 0.05). The number of neutrophils in TSG/BYG group was reduced compared with TSG/NS group from Day 18 to 22 and was reduced in MXF+STL/STL group compared with MXF+STL/NS group on Day 12 (*P* < 0.05).

As shown in Figure 5(d), the number of lymphocytes in each group did not differ throughout the course of AE-RW-COPD.

### 3.6. Cell Types in Bronchoalveolar Lavage Fluid

**Figure 6:**
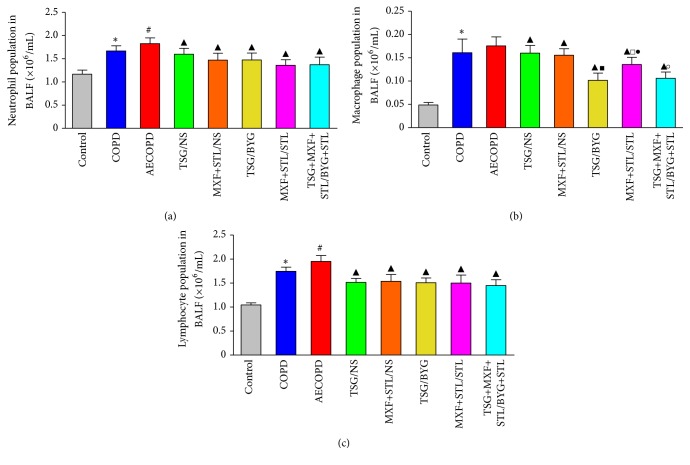
Changes in the numbers of neutrophils (a), macrophages (b), and lymphocytes (c) in the bronchoalveolar lavage fluid from rats administered sequential/nonsequential treatments. AE: acute exacerbation; BYG: Bufei Yishen Granule; COPD: chronic obstructive pulmonary disease; MXF: moxifloxacin; NS: normal saline; RW: risk window; TSG: Tongsai Granule; STL: salbutamol. *N* = 6. ^*∗*^ *P* < 0.05, versus Control group; ^#^ *P* < 0.05, versus COPD group; ^▲^ *P* < 0.05, versus AECOPD group; ^■^ *P* < 0.05, versus TSG/NS group; ^□^ *P* < 0.05, versus MXF+STL/NS group; ^●^ *P* < 0.05, versus TSG/BYG group; ^○^ *P* < 0.05, versus MXF+STL/STL group.

The numbers of neutrophils, macrophages, and lymphocytes were significantly increased in COPD group compared with Control group, and the numbers of neutrophils and lymphocytes were increased in AECOPD group compared with COPD group (*P* < 0.05) (Figure 6). Moreover, the numbers of neutrophils, macrophages, and lymphocytes were significantly decreased in the treated groups compared with AECOPD group (*P* < 0.05). The macrophage population was reduced even more in TSG/BYG and MXF+STL/STL groups compared with TSG/NS and MXF+STL/NS groups, respectively, whereas the macrophage counts in TSG+MXF+STL/BYG+STL and TSG/BYG groups were lower than in MXF+STL/STL group (*P* < 0.05).

### 3.7. C-Reactive Protein and Serum Amyloid A Levels in Serum

**Figure 7:**
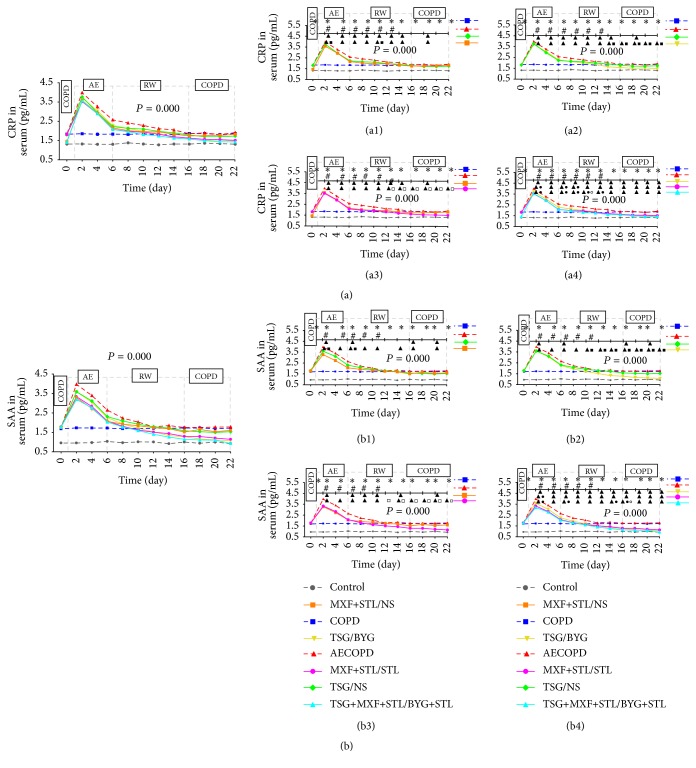
Temporal changes in C-reactive protein (CRP) (a) and serum amyloid A (SAA) levels (b) in rats administered sequential/nonsequential treatments. Panels (a1)–(a4) and (b1)–(b4) from panels (a) and (b) indicate the comparisons of the sequential and nonsequential treatment with traditional Chinese medicine, Western medicine, or integrated medicines, respectively. AE: acute exacerbation; BYG: Bufei Yishen Granule; COPD: chronic obstructive pulmonary disease; MXF: moxifloxacin; NS: normal saline; RW: risk window; TSG: Tongsai Granule; STL: salbutamol. *N* = 6. Repetitive measurement deviation analysis of CRP and SAA levels: *P* = 0.000. ^*∗*^ *P* < 0.05, versus Control group; ^#^ *P* < 0.05, versus COPD group; ^▲^ *P* < 0.05, versus AECOPD group; ^■^ *P* < 0.05, versus TSG/NS group; ^□^ *P* < 0.05, versus MXF+STL/NS group; ^●^ *P* < 0.05, versus TSG/BYG group; ^○^ *P* < 0.05, versus MXF+STL/STL group. Bacterial challenge was performed on Day 1.

As shown in Figure 7, CRP and SAA levels in COPD groups were higher than in Control group from Day 0 to Day 22 (*P* < 0.05). After challenge with the KP solution, they were highly elevated in AECOPD group on Day 2, sharply decreased on Days 4 and 6, presented a steady recovery trend in the subsequent days, and reverted to the baseline levels of COPD group on Day 16 (*P* < 0.05). CRP and SAA levels in the treated groups were lower than those in AECOPD group on Day 2, rapidly decreased over the next 4 days, changed steadily in the subsequent days, and were restored to the levels in COPD group or further decreased on Day 10–Day 14. On Day 2, the CRP and SAA levels in MXF+STL/NS group were reduced compared with TSG/NS group, and they were even lower in MXF+STL/STL and TSG+MXF+STL/BYG+STL groups compared with TSG/BYG group (*P* < 0.05). During the RW and COPD periods, the CRP and SAA levels in MXF+STL/NS group were significantly reduced compared with TSG/NS group on Day 10 and Day 6 (*P* < 0.05), and they were significantly reduced in the 2 sequential treatment groups, TSG/BYG and MXF+STL/STL, compared with TSG/NS group and MXF+STL/NS groups from Day 12–Day 22, respectively. In particular, the CRP levels in TSG+MXF+STL/BYG+STL group were much lower than those in TSG/BYG and/or MXF+STL/STL groups on Days 6–12, whereas the SAA levels on Day 6, Day 14, and Day 16 were significantly decreased (*P* < 0.05).

### 3.8. Inflammatory Factors in Serum and Bronchoalveolar Lavage Fluid

**Figure 8:**
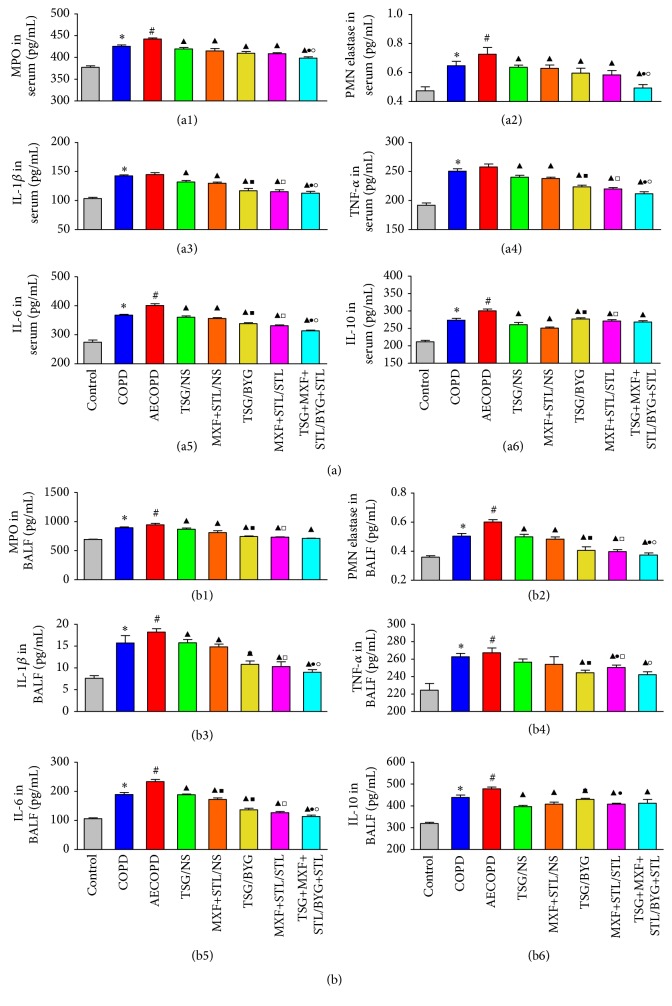
Changes in MPO (a1, b1), PMN elastase (a2, b2), IL-1*β* (a3, b3), TNF-*α* (a4, b4), IL-6 (a5, b5), and IL-10 (a6, b6) levels in the serum (a) and BALF (b) from sequentially/nonsequentially treated COPD rats. AE: acute exacerbation; BYG: Bufei Yishen Granule; COPD: chronic obstructive pulmonary disease; IL: interleukin; MPO: myeloperoxidase; MXF: moxifloxacin; NS: normal saline; PMN: polymorphonuclear; RW: risk window; TNF: tumor necrosis factor; TSG: Tongsai Granule; STL: salbutamol. *N* = 6. ^*∗*^ *P* < 0.05, versus Control group; ^#^ *P* < 0.05, versus COPD group; ^▲^ *P* < 0.05, versus AECOPD group; ^■^ *P* < 0.05, versus TSG/NS group; ^□^ *P* < 0.05, versus MXF+STL/NS group; ^●^ *P* < 0.05, versus TSG/BYG group; ^○^ *P* < 0.05, versus MXF+STL/STL group.

MPO, PMN elastase, IL-1*β*, TNF-*α*, and IL-6 and IL-10 levels in serum and BALF of COPD group were significantly increased compared with Control group (*P* < 0.05) (Figure 8). Similarly, MPO, PMN elastase, and IL-6 and IL-10 levels in serum and BALF and the IL-1*β* and TNF-*α* levels in BALF of AECOPD group were much higher than those in COPD group (*P* < 0.05). Serum and BALF levels of all inflammatory factors in the treated groups were reduced compared with those of AECOPD group (*P* < 0.05).

For the treated groups, serum IL-1*β*, TNF-*α*, IL-6, MPO, and PMN elastase levels and BALF MPO and PMN elastase levels were decreased in TSG/BYG and MXF+STL/STL groups compared with those in TSG/NS and MXF+STL/NS groups (*P* < 0.05). Serum levels of the above-mentioned indicators and BALF PMN elastase, IL-1*β*, and IL-6 were significantly reduced in TSG+MXF+STL/BYG+STL group compared with TSG/BYG and MXF+STL/STL groups (*P* < 0.05). BALF TNF-*α* in MXF+STL/STL group was significantly higher than those in TSG/BYG and TSG+MXF+STL/BYG+STL groups (*P* < 0.05). Serum IL-10 in TSG/BYG and MXF+STL/STL groups were significantly increased compared with TSG/NS and MXF+STL/NS groups, respectively (*P* < 0.05), and BALF IL-10 in TSG/BYG group was significantly increased compared with TSG/NS and MXF+STL/STL groups (*P* < 0.05).

### 3.9. Pulmonary Morphology and Morphometry

**Figure 9:**
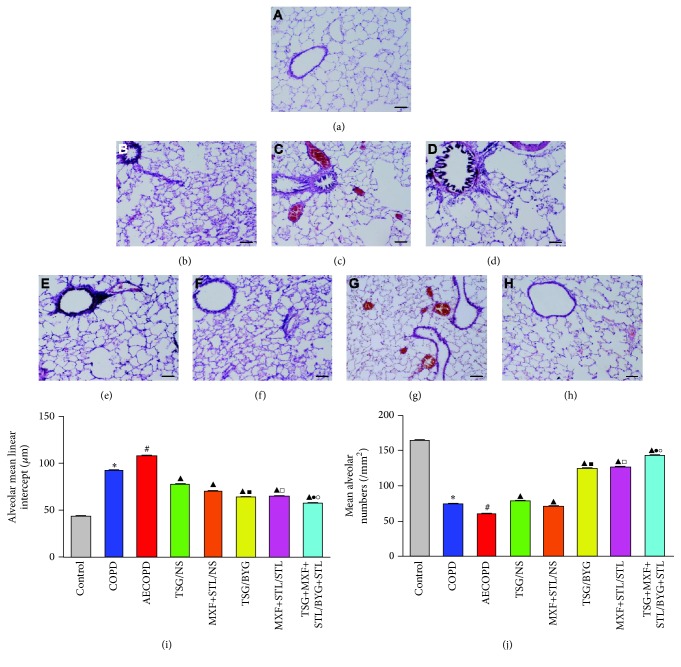
Representative images of the pathology in lung tissues from rats administered sequential/nonsequential treatments. (a) Control group; (b) chronic obstructive pulmonary disease (COPD) group, (c) acute exacerbation of COPD (AECOPD) group; (d) TSG/NS group; (e) MXF+STL/NS group; (f) TSG/BYG group; (g) MXF+STL/STL group; (h) TSG+MXF+STL/BYG+STL group. H&E stained, magnification: ×100. (i) Mean linear intercept (MLI); (j) mean alveolar numbers (MAN). AE: acute exacerbation; BYG: Bufei Yishen Granule; COPD: chronic obstructive pulmonary disease; MXF: moxifloxacin; NS: normal saline; RW: risk window; TSG: Tongsai Granule; STL: salbutamol. *N* = 6. ^*∗*^ *P* < 0.05, versus Control group; ^#^ *P* < 0.05, versus COPD group; ^▲^ *P* < 0.05, versus AECOPD group; ^■^ *P* < 0.05, versus TSG/NS group; ^□^ *P* < 0.05, versus MXF+STL/NS group; ^●^ *P* < 0.05, versus TSG/BYG group; ^○^ *P* < 0.05, versus MXF+STL/STL group.

No obvious pathological impairments were observed in Control group (Figure 9(a)). Marked chronic bronchiolar and pulmonary inflammation and obstruction, airway wall thickening and hyperplasia, and alveolar destruction were observed in COPD rats (Figure 9(b)), particularly in those suffering from acute exacerbation (Figure 9(c)). However, the impairments were reduced to different degrees in the treated groups (Figures 9(d), 9(e), 9(f), 9(g), and 9(h)), of which TSG/BYG, MXF+STL/STL, and TSG+MXF+STL/BYG+STL groups had fewer impairments (Figures 9(f), 9(g), and 9(h)). As shown in Figures 9(i) and 9(j), MLI in COPD group was significantly increased compared with Control group, whereas MAN was significantly decreased (*P* < 0.05). MLI in AECOPD group was even higher than in COPD group, and MAN was lower than in COPD group (*P* < 0.05). Furthermore, all of the treated groups had reduced variations compared with AECOPD group (*P* < 0.05). MLI in TSG/BYG and MXF+STL/STL groups was significantly reduced compared with TSG/NS and MXF+STL/NS groups, respectively, and MLI in TSG+MXF+STL/BYG+STL group was further decreased compared with TSG/BYG and MXF+STL/STL groups (*P* < 0.05). MAN in TSG/BYG and MXF+STL/STL groups was elevated compared with TSG/NS and MXF+STL/NS groups and was even higher in TSG+MXF+STL/BYG+STL group than in TSG/BYG and MXF+STL/STL groups (*P* < 0.05).

## 4. Discussion

This is the first study to discuss the therapeutic effects of sequential treatments with Tongsai and Bufei Yishen Granules during the AE-RW period in a rat model of AECOPD. The major findings of this study indicate that sequential treatments in the AE and RW phases improved pulmonary function, reduced systemic inflammation, and shortened the recovery time, especially the sequential treatment with the combination of Chinese and Western medicines.

In TCM, COPD belongs to the category of FEIZHANG disease, which is characterized as deficient root and excessive superficial throughout the course of disease. The lungs and kidneys govern innate and postnatal qi of the body, and the deficiency of the lung and kidney qi will result in breathlessness, cough, and sputum production and is considered as one of the most common syndromes, the lung-kidney qi deficiency syndrome, in the stable phase of COPD. During the acute exacerbation of COPD, pathogenesis is mainly considered an invasion of external pathogenic factors, including wind-cold and wind-heat, which can develop into phlegm-dampness and phlegm-heat syndromes; phlegm-heat is the most important syndrome in the AE stage. The main features of phlegm-heat syndrome are fever, cough, and yellow/white sticky phlegm production [19]. Thus, we treated the COPD rats with a 5-day wind-heat exposure before bacterial challenge to mimic the syndrome of phlegm-heat. During the risk window, syndrome of intermingled deficiency and excess is the main pattern of pathogenesis, which is characterized by reduced phlegm-stasis complicated by the deficiency of lung-kidney qi [17]. Therefore, we treated the AE rats with Tongsai and Bufei Yishen Granules to clear the heat and expel the phlegm in the AE phase and reinforce the lung-kidney qi in the RW phase. According to our previous study, the AE phase lasts for approximately 5 days after* Klebsiella pneumoniae* challenge, followed by an approximately 10-day RW phase [14, 15]. To ensure that all rats were sacrificed in the stable phase of COPD, we stopped the administration at 7 days after RW, on Day 22.

Acute exacerbations of COPD are often followed by subsequent clinical pulmonary deterioration, which is associated with fever and a decrease in lung function, particularly in patients with frequent exacerbations [32]. Additionally, previous studies have demonstrated that COPD exacerbations are mainly associated with aggravated airway inflammation, such as increased numbers of inflammatory cells, including WBCs and neutrophils, and increased levels of acute inflammatory biomarkers, including IL-1*β*, IL-6, TNF-*α*, IL-10, CRP, and SAA [8, 11, 33–36]. Neutrophils are the predominant effector cells activated during an acute inflammatory response, and the levels of relevant MPO and PMN elastase are also elevated [8, 11, 37, 38]. Currently, CRP and SAA levels are the most common indicators used to assess systemic inflammation and curative effects [33, 39] because they show similar variation tendencies [40]. Clinical reports indicate that 24 hours after AE, pulmonary function decreases whereas the number of WBCs and neutrophils and CRP and SAA levels increase. Pulmonary function was significantly improved 72 h after patients received medication but did not fully recover until 40 days following infection [9, 10]. Pulmonary function tests, including FVC, FEV0.3, FEV0.3/FVC, and PEF, were also decreased in similar manners in COPD rat model, whereas the levels of the above-mentioned inflammatory factors were increased [41–44].

Our data indicate that body temperature and inflammatory status, including the numbers of WBCs, neutrophils, and monocytes and CRP and SAA levels, were markedly elevated in the AE rats 24 h postbacterial challenge and decreased over the next 4–6 days, whereas the PEF decreased. All of the above-mentioned targets were improved in the treated groups at different levels, and the recovery times were shortened to 2–4 days, especially with the sequential treatment with integrated Chinese and Western medicines. Tongsai Granule and/or moxifloxacin combined with salbutamol improved lung function and decreased systemic inflammation in the AE phase.

In the RW phase, all of the above-mentioned biomarkers were markedly decreased in the treated groups compared with AECOPD rat; recovery time was also decreased. PEF and inflammatory biomarkers were lower in the sequential treatment groups, such as TSG/BYG and MXF+STL/STL treatments, than in nonsequentially treated groups at different time points from Day 12 to Day 22. The recovery times were reduced from 10 days to 4–6 days in the sequentially treated groups compared with 6–8 days in the nonsequentially treated groups. The concentrations of IL-6, IL-8, IL-10, TNF-*α*, and CRP in plasma could be used for calculating the degree and process of systemic inflammation [45]. After sacrifice and 7 days after treatment, serum and BALF inflammatory factors levels, such as IL-1*β*, IL-6, TNF-*α*, IL-10, MPO, and PMN elastase, were decreased in the sequentially treated groups compared with the nonsequentially treated groups, especially in TSG+MXF+STL/BYG+STL group. These results indicate that the treatments could reduce the systemic inflammation, and the curative effects of the sequential treatments are better than the nonsequential treatments, especially the combination of TCM and Western medicines. Moreover, as an anti-inflammatory cytokine, the value of IL-10 in sequential treatment was higher than nonsequential groups, and it was even higher in BALF in TCM sequential treatment group than Western medicine group, which may imply that TSG/BYG had greater anti-inflammatory capability.

Histopathologically, chronic bronchitis, airway obstruction, alveolar structure destruction, and emphysema are the main pulmonary impairments in COPD patients. In this study, marked inflammatory cell infiltration, bronchiolar stenosis, and alveolar expansion and destruction were observed in the COPD and AECOPD rats and were improved in the treated groups at different levels. MLI and MAN, size of the alveolar cavity, and density of alveoli [31] also indicated that the level of emphysema was greater in COPD rats than in controls, particularly in AECOPD rats. All the 5 treatments alleviated emphysema, but the sequential integrated Chinese and Western medicine treatments showed a better response.

## 5. Conclusions

Sequential treatments with Tongsai and Bufei Yishen Granules in the AE-RW period can alleviate inflammation and shorten the recovery time in AECOPD rats, and sequential, integrated TCM and Western medicine treatments have more beneficial effects than TCM or Western medicine alone. This study may provide a basis for further research and the clinical applications of sequential treatments.

## Figures and Tables

**Table 1:** Protocol for treatments during the acute exacerbation and risk window phases in COPD rats.

Group	AE phase				RW phase		
	(Day −1, 0, Day 2 to Day 6)				(Day 7 to Day 16)		
	NS	TSG	MXF	STL	NS	BYG	STL
Control	+	−	−	−	+	−	−
COPD	+	−	−	−	+	−	−
AECOPD	+	−	−	−	+	−	−
TSG/NS	−	+	−	−	+	−	−
MXF+STL/NS	−	−	+	+	+	−	−
TSG/BYG	−	+	−	−	−	+	−
MXF+STL/STL	−	−	+	+	−	−	+
TSG+MXF+STL/BYG+STL	−	+	+	+	−	+	+

Note: +: treated with this medicine; −: not treated with this medicine. AE: acute exacerbation; AECOPD: acute exacerbation of chronic obstructive pulmonary disease; BYG: Bufei Yishen Granule; COPD: chronic obstructive pulmonary disease; MXF: moxifloxacin; NS: normal saline; RW: risk window; TSG: Tongsai Granule; STL: salbutamol.

**Table 2:** Mortalities of the rats in each group.

Group	*N*	Number of deaths	Mortality (%)
Control	8	0	0
COPD	8	1	12.5
AECOPD	8	2	25^*∗*^
TSG/NS	8	0	0
MXF+STL/NS	8	0	0
TSG/BYG	8	0	0
MXF+STL/STL	8	0	0
TSG+MXF+STL/BYG+STL	8	0	0

Note: AE: acute exacerbation; AECOPD: acute exacerbation of chronic obstructive pulmonary disease; BYG: Bufei Yishen Granule; COPD: chronic obstructive pulmonary disease; MXF: moxifloxacin; NS: normal saline; TSG: Tongsai Granule; STL: salbutamol. ^*∗*^ *P* < 0.05 versus Control group.

## Abbreviations

AE: Acute exacerbation
BALF: Bronchoalveolar lavage fluid
BYG: Bufei Yishen Granule
CFU: Colony forming units
COPD: Chronic obstructive pulmonary disease
CRP: C-reactive protein
FEV0.3: Forced expiratory volume in 0.3 s
FVC: Forced vital capacity
KP: * Klebsiella pneumonia*
IL: Interleukin
MPO: Myeloperoxidase
MXF: Moxifloxacin
PEF: Peak expiratory flow
PMN: Polymorphonuclear
RW: Risk window
SAA: Serum amyloid A
STL: Salbutamol
TCM: Traditional Chinese medicine
TNF: Tumor necrosis factor
TSG: Tongsai Granule.
